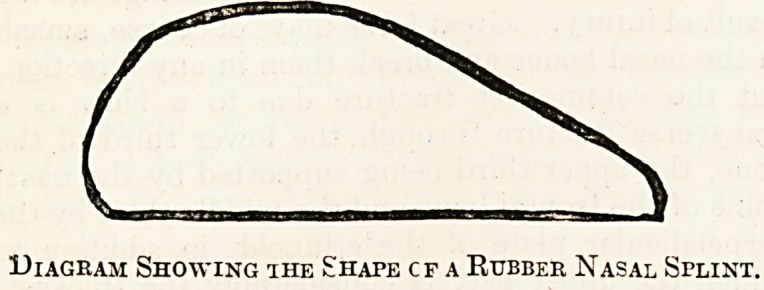# Fractures of the Nose

**Published:** 1909-08-21

**Authors:** 


### Laryngology and Rhinology.

### FRACTURES OF THE NOSE.

Injuries of the nose, with fracture of the cartilage or bone, are of considerable importance in practice, for they unite so rapidly that, unless promptly treated, they are almost certain to result in permanent deformity. It is important to remember that, while only severe fractures cause external deformity, a marked deflection of the septum may result from comparatively slight trauma, and may produce nasal obstruction and all its sequelæ although accompanied by no change in shape of the external parts of the nose, and that this fracture of the septum is to be detected only by intranasal examination. Again, it must not be forgotten that deformities resulting from such fractures are almost impossible to correct satisfactorily.

The part most frequently damaged is the triangular cartilage of the septum; next in frequency, though a considerable way behind, come the nasal bones; the perpendicular plate of the ethmoid is seldom broken, and the vomer practically never.

### The Triangular Cartilage.

The triangular cartilage is most frequently broken in one or other of two directions, either about an oblique line parallel to the upper border of the vomer, or about a more or less vertical line well forward in the nose near the junction of the vestibule with the cavity of the nares proper. In the latter case the septal projection is situated at the narrowest part of the nares, which corresponds to the groove behind the ala nasi, and is therefore the more obstructive; frequently that portion of the septum in front of the fracture is so deflected as to look almost forwards, and to be quite noticeable to the patient and his friends. The oblique line of fracture, parallel to the edge of the vomer, produces the well-known ridge or " spur " running from the floor of the nose just behind the vestibule in a direction backwards and upwards. Fractures of the septum often leave behind very considerable thickening, so that obstructive knots or bosses project into both nares. Not infrequently the cartilage is fractured in both of the positions described above, and the entire area between the two lines of fracture is then pushed over to one side. When the cartilage is much deflected, its anterior edge is often dislocated from between the inner limbs of the two lower lateral cartilages, and projects into the nostril on the side opposite to that towards which the main part of the cartilage is displaced; that is, like the edge of a cup, it appears on the side of the concavity. In addition to these fractures, the septal cartilage may be dislocated from its attachment to the vomer; this accident gives rise to the appearance of an oblique cartilaginous ridge upon the side of displacement and, on the opposite side but at a lower level, a similar ridge due to the projection of the edge of the vomer.

### The Nasal Bones.

While many deflections of the septum are, in all probability, not traumatic, it may be said that all cases associated with deformity of the bridge are the result of injury. Great force may, of course, smash in the nasal bones and break them in any direction, but the commonest fracture due to a blow is a transverse fracture through the lower third of the bone, the upper third being supported by the nasal spine of the frontal bone and the middle third by the perpendicular plate of the ethmoid, in addition to which the upper part is considerably the thickest. One nasal bone only is generally broken, while the septal cartilage is also fractured and displaced towards the side opposite to the fractured nasal bone. This gives rise to the very characteristic deformity, for the depression of the broken fragment causes the entire bridge of the nose to appear deflected towards the opposite side together with the tip of the nose, which is carried over by the deflected septal cartilage. If the septum is not much distorted, the bridge of the nose appears to curve to one side and return towards the middle line at the tip. It is in association with fracture of the nasal bones that fractures of the perpendicular plate of the ethmoid occur, but any obvious fracture or displacement in this region is decidedly rare.

### Treatment.

Fractures of the nose are in all probability always compound, but they nevertheless unite with remarkable rapidity, and septic infection and necrosis are very uncommon. It is a peculiar fact that hæmatoma of the septum is almost unknown in adults, but is quite common in children after injury. It is always associated with fracture of the septum so that the collection of blood bulges into both nares beneath the muco-periosteum. The proper treatment of this lesion is to open it by a free incision on one side, clear out the clot and drain the cavity freely. The last object is best effected by snipping away a little of the mucous membrane backwards from the incision, for a drainage tube will not keep in position. The little operation is easily performed under cocaine. Immediate incision is recommended for the following reasons: absorption is slow and incomplete, and leaves behind considerable obstructive thickening, suppuration is very apt to ensue, in which case there is great danger of a falling in of the bridge just below the nasal bones, causing a very unsightly deformity; and, finally, it is impossible to tell whether the fractured septum is displaced until the hæmatoma has subsided, by which time the fragments will have become firmly consolidated.

In cases of fracture with displacement, the septum must be replaced under anæsthesia as soon as possible after the injury with a pair of flat-bladed septum forceps, such as Walsham's; it is then kept in position by means of a splint. The best form of splinting is made from a piece of solid red rubber sheeting, which can be obtained in various thicknesses, boiled before use, and cut to the required size and shape at the time; the accompanying diagram shows the proper shape; the narrower end looks forwards to the nostril. It is carried into the nose on a nasal forceps until it just reaches the posterior nares, as ascertained by a finger in the

Diagram Showing the Shape of a Rubber Nasal Splint.

nasopharynx, and its anterior end should lie just within the nostril. It is usually only necessary to use a splint in one nostril, on the side of the displacement, and it should be kept in for eight to ten days. It can be kept clean by using a nasal lotion with a syringe provided with a fine nozzle which can be introduced on either side of the splint. A depressed fracture of the nasal bone can be raised by introducing a stiff probe into the nose, or by grasping it with the septal forceps, one blade in the nose and one outside, the latter being protected with rubber tubing. It is often, however, extremely difficult to keep this fragment in position if the depression tends to recur; the splint, made of rather thin rubber sheeting, and of a somewhat deeper shape than usual, is frequently effective. Gauze packing should be avoided as it quickly becomes foul, and shields moulded to fit over the bridge are generally useless, as they have no power to raise the depressed fragment. It is sometimes necessary to hold the fragment up by means of a stout hare-lip pin driven through the bridge at its base, a piece of indiarubber being put over each end and held together with steady pressure by tying a silk thread over all with a figure of eight.

## Figures and Tables

**Figure f1:**